# Efficient Singlet Oxygen Monitoring in Aqueous Media Comprising a Polymer‐embedded Eu^3+^‐Complex

**DOI:** 10.1002/chem.202500943

**Published:** 2025-05-22

**Authors:** Daniel K. Dinga, María V. Cappellari, Cristian A. Strassert, Ulrich H. Kynast

**Affiliations:** ^1^ Institute for Optical Technologies Muenster University of Applied Sciences 48565 Steinfurt Germany; ^2^ Institute for Inorganic und Analytical Chemistry University of Münster Corrensstraße 28/30 48149 Münster Germany; ^3^ CiMIC, SoN CeNTech University of Münster Heisenbergstraße 11 48149 Münster Germany

**Keywords:** bacteria, lanthanides, polymers, sensors, singlet oxygen detection

## Abstract

Singlet dioxygen (^1^O_2_) plays a pivotal role as the active agent in photodynamic therapy (PDT) for cancer treatment, as well as in the photo‐inactivation of antibiotic‐resistant microbes (antimicrobial photodynamic therapy, aPDT). The ability to sensitively monitor the production and behavior of ¹O₂ following its photo‐catalytic generation is crucial for developing effective therapeutic strategies. Optical sensor molecules that respond to ¹O₂ through changes in absorption or, more sensitively, fluorescence, are suitable choices. While most monitors report ^1^O_2_ via altered absorption spectra, only few compounds respond by the onset of fluorescence, even fewer based on lanthanide luminescence. By embedding a novel lanthanide complex (Eu^3+^) into polystyrene nanoparticles (beads), we achieved close to a 500‐fold emission intensity boost in the presence of ^1^O_2_, very long decay times of up to 879 µs and unprecedented stability in acidic and basic media. Furthermore, the beads present a high‐surface charge (>+30 mV), yielding stable aqueous dispersions, which we exploited in a preliminary “proof of principle” staining experiment of (negatively charged) bacterial surfaces. The straightforward synthesis circumvents intricate preparative steps and restrictive costs. The decay characteristics furthermore pave the road to time‐gated measurements, that is, to the suppression of interfering autofluorescence from biological samples.

## Graphical abstract

A singlet dioxygen (^1^O_2_) sensitive Eu(III) complex embedded in polymer nanoparticles efficiently detects ^1^O_2_ in aqueous media by a remarkable increase in Eu^3+^ luminescence. These particles also adhere to bacteria surfaces, where the cells appear brightly stained in the presence of ^1^O_2_ enabling spectroscopic evaluation and easy detection under the microscope.

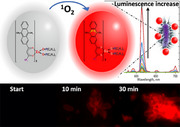

## 1 Introduction

Singlet dioxygen (^1^O_2_) is a highly reactive alter ego of the comparatively inert triplet dioxygen (^3^O_2_) and a reagent with many facets of efficacy, ranging from organic oxygenation or oxidation reactions^[^ ^1^ ^]^ to biological processes.^[^ ^2^ ^]^ In technical terms, ground state ^3^O_2_, a diradical with two unpaired electrons in oxygen's antibonding molecular π*‐orbitals (^3^Σ_g_^−^), can appear in two excited states with spin‐paired electrons either in only one of the π∗‐orbitals (^1^Δ_g_) or two electrons of opposite spin in both π∗‐orbitals (^1^Σ_g_^+^). The lower excited state ^1^Δ_g_ lies 94 kJ/Mol above the ground state, whereas ^1^Σ_g_^+^ resides at 156 kJ/mol.^[^ ^3^, ^4^ ^]^ Singlet dioxygen is one of the reactive oxygen species (ROS, O_2_^•−^, ^•^OH, ^1^O_2_ among some others^[^ ^5^ ^]^). Only to name two topical applications: the cytotoxicity of ^1^O_2_ can be exploited in both the fight against cancer and the ever‐increasing threat of antimicrobial resistance (AMR); thus, photodynamic therapy (PDT) aims at cancer, while antimicrobial photodynamic therapy (aPDT) targets microbes, pathogenic bacteria in particular. Both of them have been reviewed extensively in the past.^[^ ^6^, ^7^, ^8^, ^9^, ^10^, ^11^, ^12^ ^]^ As the sensitive detection of ^1^O_2_ rather than its generation is in focus here, we shall restrict ourselves to a brief mechanistical discussion of key steps involving its artificial genesis. Both PDT and aPDT rely on the photo‐sensitized conversion of ^3^O_2_ → ^1^O_2_. A direct photo‐conversion is forbidden quantum‐mechanically (spin, parity, and angular momentum selection rules) and at least 10^5^ times less efficient than sensitized photo‐activation^[^ ^4^, ^13^ ^]^ with appropriate photo‐sensitizers (e.g., porphyrins, phthalocyanines, methylene blue, rose bengal (RB)).^[^ ^14^, ^15^ ^]^ The photosensitizers, after the absorption of visible light into an excited singlet state, undergo an isoelectronic radiationless transition to yield a triplet state (intersystem crossing ^1^S* → ^3^T). Upon collision with ^3^O_2_, reactive oxygen species (ROS) are formed, which can subsequently react with the targets, these being the malignant cells in PDT or microbial pathogens in aPDT, respectively. If ^1^O_2_ is the useful reactant, subsequent reactions are termed type II. Of crucial importance is the lifetime of generated ^1^O_2_, as it determines the proximity required for successful reaction with the target before collisional or vibronic quenching. In water—most important for biological objects—the lifetime amounts to 3.45 µs^[^ ^16^ ^]^ resulting in a diffusion distance of approximately 200 nm.^[^ ^17^ ^]^

The ability to monitor the ^1^O_2_ evolution in various solvents under a broad range of conditions is a persisting challenge: direct measurements based on the 1270 nm phosphorescence require very sensitive, dedicated equipment due to the very low phosphorescence quantum yield (<10^−6^ in water^[^ ^18^, ^19^ ^]^), which is typically available in specialized labs only. Hence, indirect methods employing probes whose optical properties are altered substantially in contact with ^1^O_2_ are a most convenient and reasonable alternatives. Properties that have been widely exploited are absorption and fluorescence; other methods like chemiluminescence and spin signaling have been described as well.^[^ ^20^, ^21^ ^]^ Within the plethora of probes known today, reporters responding to the presence of ^1^O_2_ with an increase of a fluorescence signal (“positive probes”) rather than fading of an absorption or emission (“negative probes”) have in recent years proven to be of particular value due to superior sensitivity.^[^ ^21^, ^22^, ^23^, ^24^ ^]^ Among the positive probes, a handful of lanthanide (rare earth) complexes deserve particular attention as they readily pave the road to time‐gated emission monitoring, that is, the possibility of blotting out parasitic emissions, autofluorescence among them.^[^ ^25^ ^]^ An overview of existing Ln^3+^ probes for ROS detection also addresses other cytotoxic or signaling species (OCl^−^ and Reactive Nitrogen Species, RNS) and includes detection using EPR and MRI;^[^ ^26^ ^]^ intracellular ^1^O_2_ detection with both, rare earth and transition metal complexes has recently been reviewed also.^[^ ^27^ ^]^

**Scheme 1:**
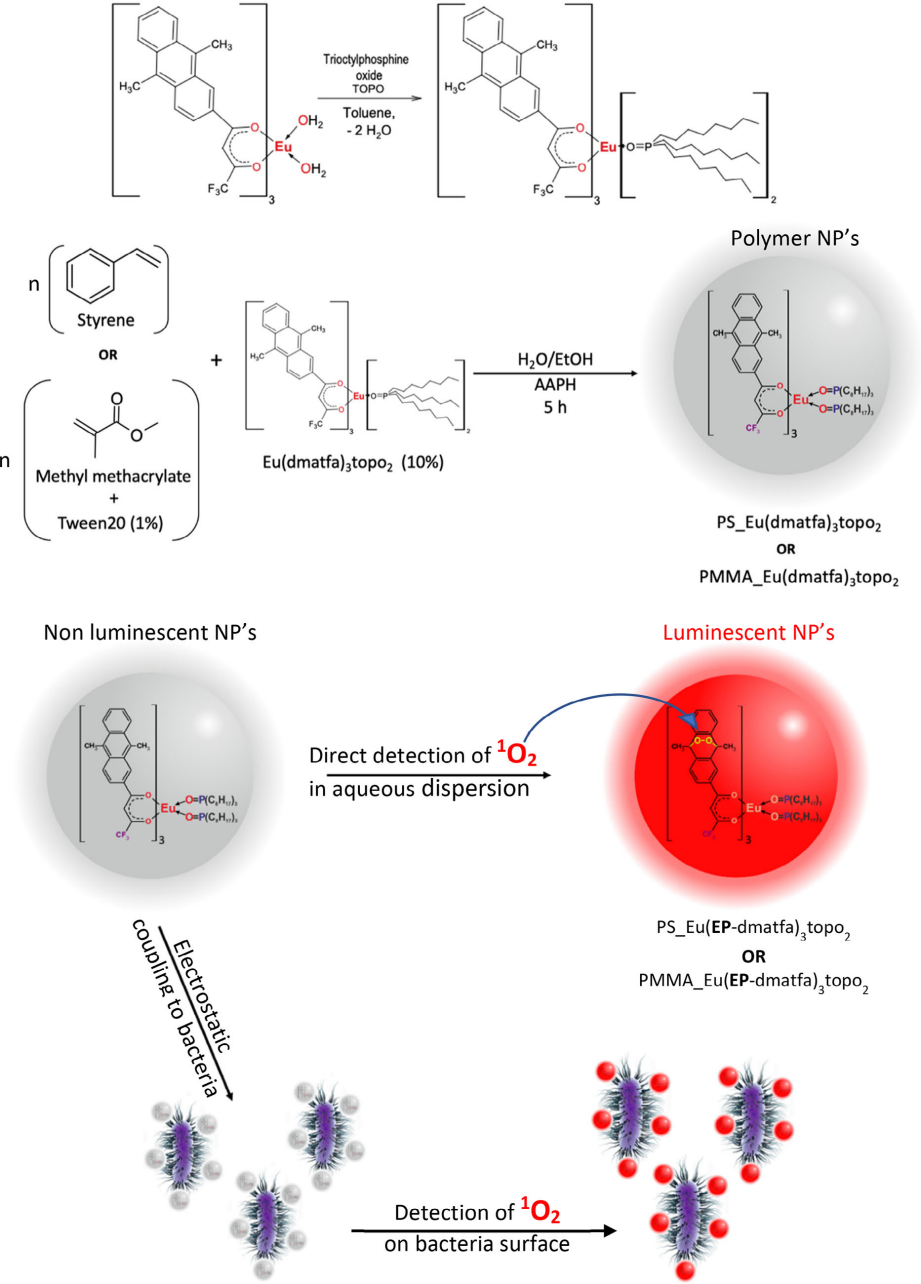
Synthesis pathway toward polymeric nanoparticles (NPs) loaded with an Eu(III) complex as ^1^O_2_ sensor, starting from the binary complex to illustrate its application in aqueous dispersions and on bacteria.

In luminescent lanthanide complexes, an antenna ligand collects incident radiation via an allowed singlet transition (^1^S_0_ → ^1^S*). By intersystem crossing, the excited singlet state relaxes into an associated triplet state (^1^S* → ^3^T), which intramolecularly transfers its energy to the trivalent rare ion (^3^T → Ln^3+^, e.g., Eu^3+^, Tb^3+^). Ideally, the return to the Ln^3+^ ground state leads to characteristic narrow emission lines of the Ln^3+^ ion. The emissive return to the Ln^3+^ ground state is parity‐forbidden, resulting in very long lifetimes of the excited Ln^3+^ ions in the micro‐second regime, which is in turn the mechanistic basis for time‐gated measurements (see, e.g., W. Horrocks and Albin^[^ ^28^ ^]^ and Bünzli and Eliseeva^[^ ^29^ ^]^). Furthermore, if the ligand ^3^T is energetically lower than the excited Ln^3+^ state, the complex will not emit, which is the case for anthracene and most phenanthrene derivatives. The key to their employment as luminescent probes is the reactivity toward ^1^O_2_. In the case of anthracenes, endoperoxides are formed, which exhibit a triplet state well above the original anthracene moiety,^[^ ^30^ ^]^ thus allowing efficient emission. One of the first complexes employing anthracene substituted ligands was reported already 20 years ago;^[^ ^31^ ^]^ a Eu^3+^ complex similar to those under discussion here could be used to detect ^1^O_2_ in mitochondria in 2015.^[^ ^32^ ^]^ Similar principles are taken advantage of in other “positive” sensors BODIPY‐DPA,^[^ ^33^ ^]^ Si‐DMA,^[^ ^34^ ^]^ commercial SOSG,^[^ ^24^ ^]^ and Aarhus Sensor Green.^[^ ^35^ ^]^ In these, the anthracene moiety is attached to an organic luminophore, whose emission is quenched by photoinduced electron transfer (PET) quenching. After ^1^O_2_‐trapping and endoperoxidation, the PET channel is blocked, and the fluorescence of the dye emerges. The lanthanide complex employed in the work presented herein is bis(trioctylphosphine oxide)‐tris(1,1,1‐trifluoro‐5‐(9,10‐dimethyl‐2‐anthryl)‐3,5‐pentanedione)europium, Eu(dmatfa)_3_(topo)_2_, and its endoperoxide bis(trioctylphosphine oxide)‐tris(1,1,1‐trifluoro‐5‐(9,10‐dimethyl‐9,10‐μ‐peroxo‐2‐anthryl)‐3,5‐pentanedione)europium, Eu(EP‐dmatfa)_3_(topo)_2_, which are both readily derived from Eu(dmatfa)_3_(H_2_O)_2_ (Scheme 1).^[^ ^30^ ^]^ As in the PET‐probes above, the 9,10‐dimethyl‐2‐anthryl‐substituent acts as the ^1^O_2_‐trap, while the trifluoro‐substitution of aromatic diketonates with Eu^3+^ is known to yield efficient rare earth complexes,^[^ ^36^ ^]^ particularly in conjunction with ancillary trioctylphosphine oxide (topo) ligands. Topo here has a twofold function: it prevents luminescence quenching by coordinating H_2_O around the Eu^3+^ coordination sphere and provides high solubility of the complex in organic solvents, styrene, and methyl methacrylate in particular.^[^ ^37^ ^]^

A general shortcoming of Eu^3+^‐ß‐diketonate complexes in aqueous media, like in biological matrices, are stability issues accompanied by efficiency loss. This was recognized at an early stage of utilizing ß‐diketonate complexes for bioassays: an elegant remedy to circumvent instability against water and concomitant bottlenecks was disclosed in a patent claiming “Fluorescent labels comprising rare earth chelates” in 1981,^[^ ^38^ ^]^ in which the incorporation of complexes into polymer beads of 100 to 150 nm is described. Thousands of complex molecules may be occluded per bead, giving enormous advantages with respect to efficiency and brightness.^[^ ^37^ ^]^ In the context of ^1^O_2_‐sensing, burying the probe in a 100 nm polymer particle may seem counterintuitive on first sight, however, our experimental results show that with its considerably prolonged lifetimes (20 µs and above) in organic matrices,^[^ ^39^, ^40^, ^41^ ^] 1^O_2_ readily achieves longer diffusion lengths.

## 2 Results and Discussion

### 2.1 Synthesis and Characterization

**Figure 1:**
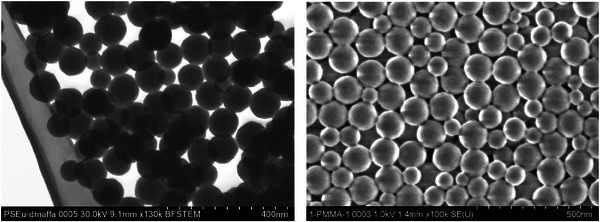
Bright Field STEM of PS_Eu (left) and SEM of PMMA_Eu NPs (right). Colloidal dispersions of 0.1 wt% at pH 7.5 were used.

**Figure 2:**
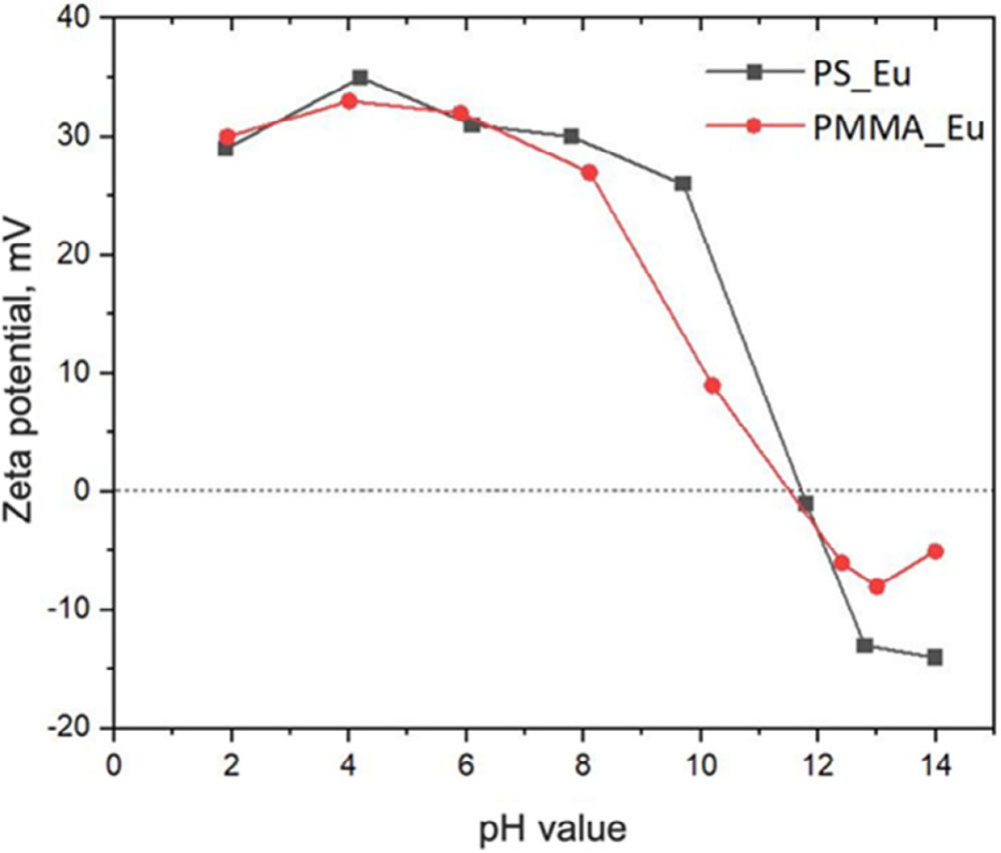
Zeta potentials of the PS_Eu and PMMA_Eu NPs at various pH levels. Colloidal dispersions of 0.01 wt% were used.

The binary europium complex diaqua‐tris(1,1,1‐trifluoro‐5‐(9,10‐dimethyl‐2‐anthryl)‐3,5‐pentanedione) europium(III) was shown to be a very effective and efficient ^1^O_2_ sensor. However, one serious drawback restricting its application in biological systems is related to its insolubility in water. To address this, the complex was incorporated into polymer beads. The aqua‐complex was coordinated with the ancillary topo ligand in order to expel water from the first coordination sphere and more importantly, to enhance the complex's solubility in organic phases, facilitating its incorporation into polymer beads during synthesis of the nanoparticles. This method has previously led to loadings of up to 2 wt% of europium complexes corresponding to approximately 3400 molecules (polystyrene) and 3860 molecules (polymethylmetacrylate, PMMA) particle.^[^ ^37^ ^]^ Higher concentrations of complex were possible, but increasingly led to agglomerated particles. The polymerization reaction generated beads containing the Eu(dmatfa)_3_topo_2_ complex, with particle sizes of about 100 nm for both polystyrene (PS_Eu) and polymethyl methacrylate (PMMA_Eu), as can be clearly seen in the TEM/SEM images (Figure 1). Both polymer beads showed a high zeta (*ζ*) potential of about +30 mV leading to stable colloidal dispersions. The positive ζ potential most likely results from the thermal decomposition products of the free radical initiator AAPH leading to amine and imine derivatives that adhere to the particle's surfaces.^[^ ^42^ ^]^ The colloidal PMMA bead dispersions, however, were observed to gradually flocculate after 48 hours due to a drop in their ζ−potential. The exact reason for this net drop in overall surface positive charge is not yet clear, as the polystyrene beads obtained from the same synthesis showed no such behavior. It is possible that the amine/imine residues are more easily expelled from the surface and hence the positive charges with them. The polymer beads form stable colloids over a wide pH ranging from 2 to 12 eventually, where the isoelectric point is observed (Figure 2).

### 2.2 Detection of Singlet Oxygen in Aqueous Dispersion

**Figure 3:**
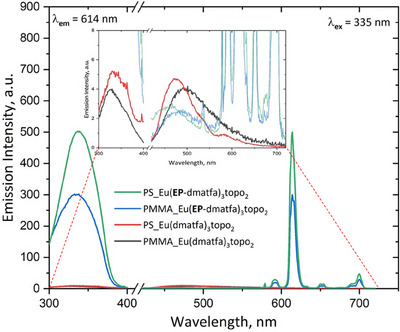
Excitation (left) and emission (right) spectra of PS_Eu and PMMA_Eu NPs before and after endoperoxidation. Colloidal dispersions of 0.01 wt% were used at pH 7.5. Endoperoxidation was attained by probe self‐sensitization, through exposure to a 3.5 W, 430 nm LED for 30 minutes. The inset provides a magnified view, showing the spectra of the probes before endoperoxidation at an appropriate scale.

**Figure 4:**
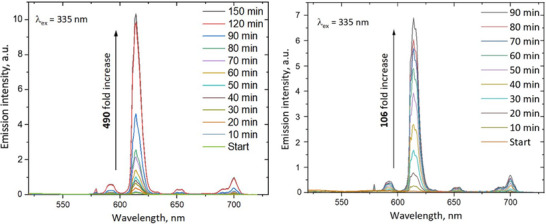
Luminescence rise of PS_Eu (left) and PMMA_Eu (right) upon continuous exposure to ^1^O_2_ generated by RB in aqueous dispersion. Colloidal dispersions of 0.01 wt% were used at pH 7.5 at an RB concentration of 5 µM. Irradiation proceeded in a photoreactor with 1.0 W 520 nm LED.

**Figure 5:**
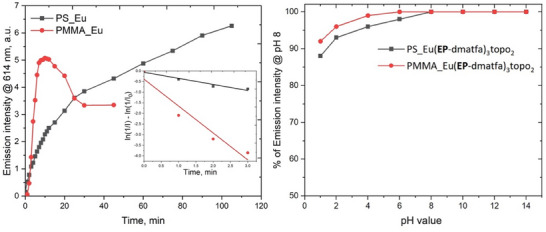
(Left) Plot of the luminescence intensity (I) of both PS/PMMA_Eu probes at 614 nm with increasing irradiation time and photosensitized exposure to ^1^O_2_. The inserted figure shows a plot of ln(1/I)‐ln(1/I_0_) versus irradiation time at pH 7.5 RB concentration of 5 µM. Irradiation was conducted in a photoreactor with a 1.5 W / 520 nm LED. (Right) Stability test of the endoperoxidized PS and PMMA_Eu probes at various pH levels. Dispersions of 0.01 wt% were used.

The PS_Eu and PMMA_Eu probes were tested for their response to ^1^O_2_ in aqueous dispersion. Before exposure to ^1^O_2_, the NPs show a broad but faint emission spectrum starting from about 430 nm tailing off around 700 nm (Figure 3), which peaks at about 470 nm for PS_Eu and 490 nm for PMMA_Eu. This emission signal is visible even after complete endoperoxidation of the probe, indicating that the polymer matrix contributes as well to some degree. After complete endoperoxidation of the complexes in the beads, the final PS/PMMA_Eu(**EP**‐dmatfa)_3_topo_2_ shows a broad excitation spectrum from about 280 nm to about 390 nm with a maximum at around 335 nm. The characteristic Eu^3+^ emission spectrum emerges as well, which overshadows the initial emission signals before the probes’ reaction with ^1^O_2_. Both probes show bi‐exponential decays (Table 1) with a major contribution of about 70% relative amplitude and a minor of 30%. This indicates that not all the complexes are in the same region of the beads, as a majority probably lies at the heart of the particles while a smaller fraction allocates closer to or within the interface. However, we can be certain that the Eu(III) complex molecules are not simply adsorbed on the polymer surface, since the shorter (the minor) decay time observed for PS_Eu(**EP**‐dmatfa)_3_topo_2_ NPs is 431 µs, which is even longer than the decay time of the endoperoxidized topo‐complex in ethanol,^[^ ^30^ ^]^ indicating that its environment is void of ‐OH vibrations. Most significantly, both probes maintain the peak Eu^3+^ emission intensity even in extremely acidic environments (Figure 5). The main decay time for the PS_Eu(**EP**‐dmatfa)_3_topo_2_ reaches up to 879 µs, which is about a third longer than that of the pure endoperoxidized topo‐complex in organic solvents. This remarkably long decay time will come in most handy in time‐gated evaluations of singlet dioxygen generation in biological systems. Both particles displayed an extraordinarily strong luminescence response to ^1^O_2_ with a 490‐fold increase in emission signal for the PS_Eu beads and over 106‐fold increase for the PMMA_Eu beads (Figure 4).

**Table 1:** Comparison of physical and spectroscopic properties of the PS_Eu and PMMA_Eu probes.

	PS_Eu(dmatfa)_3_topo_2_	PMMA_Eu(dmatfa)_3_topo_2_
Yield of polymerization	71%	30%
Particle size	97 nm	102 nm
Zeta potential, ζ	+32 mV	+31 mV
ζ stability	Months	48 h
Luminescence response to ^1^O_2_	490‐fold increase	106‐fold increase
	PS_Eu(*EP*‐dmatfa)_3_topo_2_	PMMA_Eu(*EP*‐dmatfa)_3_topo_2_
Decay time, τ	879 µs [82%] **/** 596 µs [18%]	495 µs [73%] **/** 174 µs [27%]
pH stability range	1‐12	1‐12

Once completely endoperoxidized, the PS_Eu probe shows far greater photostability compared to the PMMA_Eu probe, as the peak Eu^3+^ intensity is maintained. Photostability is particularly advantageous during prolonged light exposures like the photosensitization process itself and also the intense excitation during fluorescence microscopy. Since the Eu^3+^ species is coordinated by three antenna ligands that quench the intrinsic Eu^3+^ luminescence before peroxidation, stepwise reaction of the ligands with ^1^O_2_ does not lead to resolved, proportional intensity jumps of the Eu^3+^ signal.^[^ ^39^ ^]^ The resulting nonlinear relationship between the Eu^3+^ emission intensity and the reaction product concentration renders the determination of the reaction rate constant of the PS‐Eu and PMMA_Eu probes with ^1^O_2_ difficult. This is also compounded by the most likely random distribution of Eu‐complex within the polymer particles and, given the possibly of different environments around the Eu(III) complex (surface‐near vs. deep within the particle), the rate of reaction with ^1^O_2_ (here inexactly monitored by the increase in Eu^3+^ signal) does not follow any standard rate reaction law, hence, the kinetics cannot be easily fitted into any model.

Consequently, a simple comparison of the rate of the emission signal's increase was conducted as an attempt to compare the reactivity/sensitivity of both probes (Figure 5). For a sketchy quantitative comparison, the first 3 minutes of the reactions were assumed to follow first‐order rate kinetics; hence, the PMMA_Eu probe was estimated to be at least 10 times faster (more sensitive) in the reaction with ^1^O_2_. The higher rate suggests that the PMMA polymer matrix is more permeable to ^1^O_2_ compared to the PS matrix.

The PMMA_Eu probe, once it reaches its peak intensity, shows a drop in the emission signal by about 20% before leveling off, which may be due to coordination of the RB via its COO^‐^ moiety to the Eu^3+^ ion, displacing some of the antenna ligands. This is presumed to be the case, since the sudden drop in emission signal is not observed during self‐sensitization (irradiation without RB).^[^ ^30^ ^]^ Once the beads were exposed to ^1^O_2_ to complete endoperoxidation, the resulting luminescent dispersion was exposed to different pH environments. Remarkably, the luminescence intensity was stable across all pH ranges even after 24 hours; with only about a 10% drop in intensity occurring at the extreme acidity of pH 1.

Given that probes based on the reaction of the anthryl moiety are specific in their response to ^1^O_2_, it is clear that the incorporation of the complexes into the PS and PMMA NPs leads to stable probes, which are effective in the detection of ^1^O_2_ in aqueous dispersion at all pH ranges, leading to a more stable luminescent species than its pristine counterpart.

### 2.3 Detection of Singlet Oxygen on Bacteria Cells

**Figure 6:**
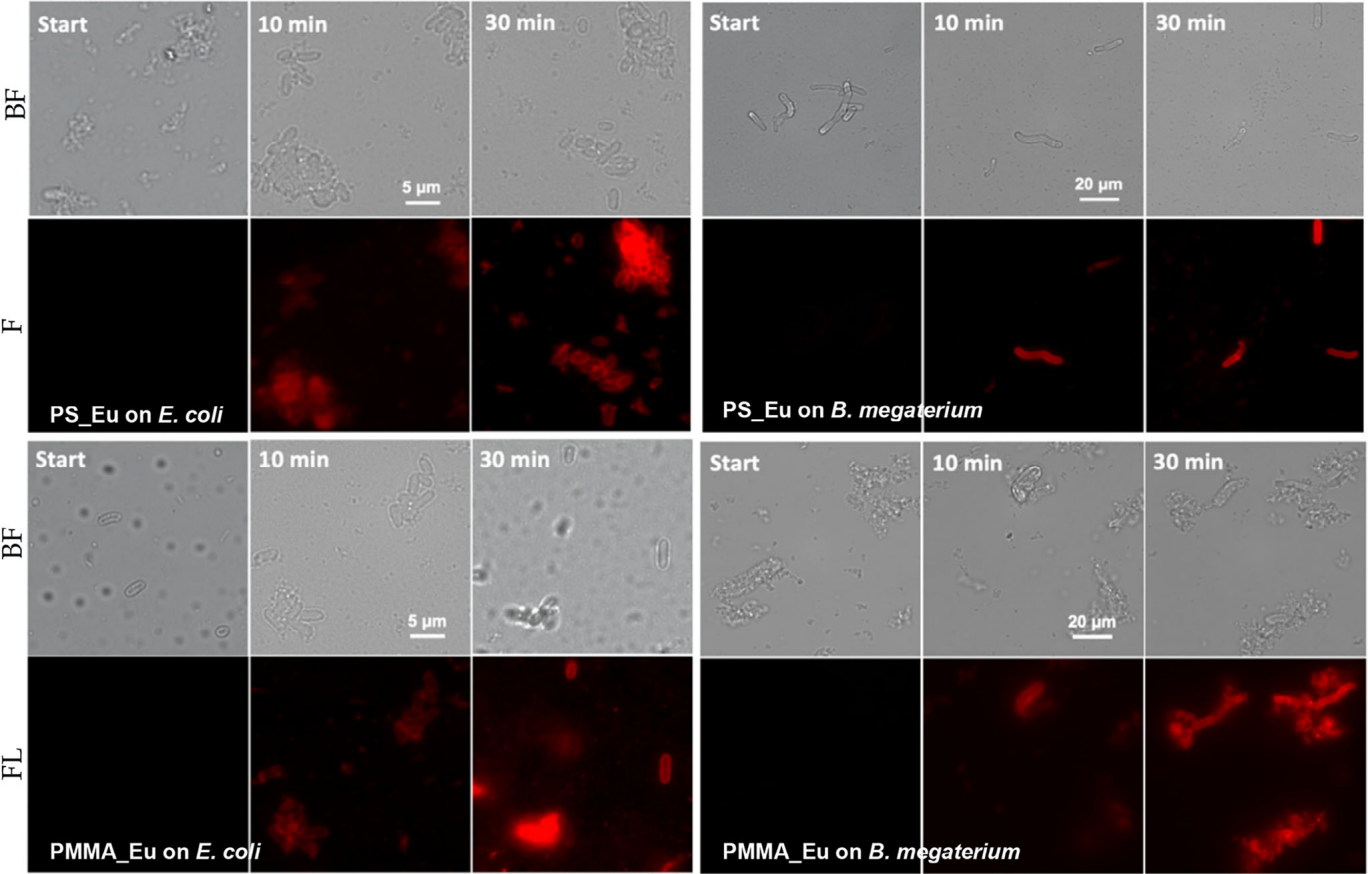
Bright field and fluorescence microscope images of the *E. coli* and *B. megaterium* bacteria containing PS‐Eu (top rows) and PMMA_Eu (bottom rows) beads attached to their surface. The fluorescence images show the gradual increase in red emission from the labelled bacteria cells on prolonged exposure to ^1^O_2_. The test dispersions contained about 10^7^ bacteria cells/mL with roughly 10^4^ beads per bacteria cell (see Supporting Information for calculation) and 5 µM RB, irradiated for up to 30 minutes using a 1.0 W LED in the photoreactor.

Due to the high positive zeta potential of the beads, they readily attach electrostatically to the negative bacterial surfaces. By observing the resulting dispersions under the microscope, the beads appeared bound to both Gram‐negative (*Escherichia coli*) and Gram‐positive (*Bacillus megaterium*) bacteria, the latter chosen for their large size and their ability to sporulate (Figure 6). The adsorption of the beads to the bacteria cell walls was superior for the PS beads, with most of the bacteria cells in dispersion surrounded by polymer beads. Both, PS_Eu and PMMA_Eu, led to a certain degree of agglomeration of the bacteria; however, this seems to be directly proportional to the concentration of beads in the dispersion. The PMMA_Eu beads nonetheless seem to be particularly pernicious in this regard, although more experiments may be needed to establish the optimum concentration ratio of beads/bacteria for best results. Once exposed to ^1^O_2_ (produced by dissolved RB), a gradual increase in the red emission signal can be seen around the bacteria, with the cells moving from pale red to bright red indicating the presence of ^1^O_2_ on the bacteria surface, since the beads themselves are too large for bacterial uptake.

This method, in conjunction with an intracellularly located probe could very well be used to differentiate between ^1^O_2_ generated outside cells versus ^1^O_2_ generated within the bacteria; we are currently elaborating the detection of ^1^O_2_ inside bacteria cells using similar rare earth complexes as probes. After prolonged bright‐field exposure, the PMMA_Eu probe gradually lost its luminescence intensity; however, the luminescence intensity of the PS_Eu remained intact.

## 3 Conclusion

We present a straightforward method for the incorporation of lanthanide complex‐based ^1^O_2_ probes into polystyrene and polymethylmethacrylate NPs for the detection of ^1^O_2_ in aqueous dispersions and on bacteria. This approach stands out due to three key advantages: (I) Superior photophysical properties with an unprecedented, 490‐fold gain in luminescence intensity upon ^1^O_2_ exposure along with an exceptionally long excited state lifetime of up to 879 µs for the PS‐hybrids, grants ultimate ^1^O_2_ sensing capacity and opens the path to time‐gated, background‐free detection. The more reactive PMMA‐hybrids still exhibit a 106‐fold intensity gain. (II) The beads form stable colloids that can detect ^1^O_2_ and maintain their brightness in a broad pH range. Hence, the performance of the beads will be widely independent from their aqueous environment, as needed for biological matrices. (III) The high positive ζ potential enables coulombic attraction and proximity to negative surfaces, as here demonstrated for bacteria. Decoration with amines also allows direct covalent coupling to numerous biologically relevant groups, targeting diverse cell surfaces, which is the object of ongoing investigations. In brief, these probes hold the potential to acquire a pole position in the detection of ^1^O_2_, with a direct link to the antimicrobial toolbox.

## 4 Experimental Section/Methods

### Synthesis of the Eu(dmatfa)_3_topo_2_ complexes

The synthesis of the binary europium complex [Eu(dmatfa)_3_(H_2_O)_2_] has been described in our previous work.^[^ ^30^ ^]^ Once the binary complex was obtained, equimolar amounts of the complex and the ancillary ligand topo were dissolved in toluene and refluxed for 3 hours. The solution was then cooled, and toluene removed by rotary evaporation to obtain the oily ternary complex Eu(dmatfa)_3_topo_2_. The complex was characterized by IR and elemental analysis (see Supporting Information)

### Synthesis of PS and PMMA beads

To a 100 mL 3‐neck flask, 35 ml of dist. H_2_O, and 17.5 mL of ethanol were added and heated at 40 °C for 30 minutes. Then, 0.8 mL of styrene/methyl methacrylate containing 12 wt% Eu(dmatfa)_3_(topo)_2_ complex and 1% Tween20 were added dropwise under ultrasonication. After further ultrasonification for 20 minutes, the reaction mixture was stirred under an N_2_ flow and a condenser at 90 °C for 1 hour, after which 0.1 g of AAPH initiator (2,2′‐azobis(2‐amidinopropane)dihydrochloride) were added and left to polymerize for 5 hours (4 hours for PMMA). Upon completion, the reaction mixture was left to cool to room temperature, followed by centrifugation to isolate the polymer beads; finally, the beads were centrifuged and washed three times with an ethanol/water mixture and redispersed in dist. H_2_O to give stable dispersions. The yield determined by gravimetric analysis was 71% (30% for PMMA) after overnight drying at 60 °C in vacuum.

### Singlet dioxygen sensing with PS_Eu and PMMA_Eu probes

To 1.8 mL of 0.1 wt% polymer bead dispersion in distilled water, 0.2 mL of 40 µM RB solution in distilled water was added. This mixture was stirred, and the emission and excitation spectra taken at regular intervals of irradiation (of theRB contained therein) in a photoreactor. The power was set to 0.5 W using a 525 LED and a 550 long‐pass filter. For the measurement of the decay time, the final endoperoxidized product of the probe was generated by self‐sensitization of the probe in the photoreactor without a filter, until no additional Eu^3+^ emission intensity increase was observed. Subsequently, the decay times were measured.

### Bacteria‐bead interaction

The bacterial cultures were taken from tryptic soy agar plates and inoculated into tryptic soy broth (caso bouillon, Merck KGaA, Darmstadt, Germany) to grow overnight. The bacteria were then centrifuged and redispersed in 50 mM HEPES buffer (pH 7.5) of above 99.5% purity as purchased from Sigma–Aldrich. The approximate numbers of bacteria in the samples were calculated using the optical density of the dispersion at 600 nm. The density was measured on a Specord 200 Plus UV‐visible spectrophotometer (Analytik Jena) using 1×1 cm acrylic cuvettes. Bacterial dispersions in HEPES buffer (2.4 × 10^8^ cells mL^−1^) were first incubated with polymer bead dispersion (final loading of 0.5 wt%) for 1 hour, followed by the addition of 200 µL of 40 µM RB solution in distilled water. Next, the mixture was added to 1800 µL of the bacteria/polymer bead dispersion, which was further incubated with agitation for 5 minutes. Finally, the dispersion was viewed under the microscope and irradiated at various time intervals in a photoreactor with a 0.5 W / 525 nm LED (with a 550 nm longpass filter).

## Conflict of Interests

The authors declare no conflict of interest.

## Supporting information



Supporting Information

## Data Availability

The data that support the findings of this study are available from the corresponding author upon reasonable request.
