# Supplementary material for: Efficient Singlet Oxygen Monitoring in Aqueous Media Comprising a Polymer‐embedded Eu3+‐Complex
Source: Chemistry. 2025 May 22;31(35):e202500943. doi: 10.1002/chem.202500943 (PMC12188158; doi:10.1002/chem.202500943)
Supplement: Supplementary file 1 — Supporting Information [file CHEM-31-e202500943-s001.docx]

**Efficient Singlet Oxygen Monitoring in Aqueous Media
Comprising a Polymer-imbedded Eu^3+^-Complex**

*aniel K. Dinga^1,2^, María V. Cappellari^2^, Cristian Strassert^2*^ and Ulrich Kynast*^1^*

^1^ Institute for Optical Technologies, Muenster University of Applied Sciences, 48565 Steinfurt, Germany

^2^Institute for Inorganic und Analytical Chemistry, University of Münster, Corrensstraße 28/30, 48149 Münster, Germany

CiMIC, SoN, CeNTech, University of Münster, Heisenbergstraße 11, 48149 Münster, Germany

*Email: ca.s@uni-muenster.de

Keywords: Singlet Oxygen Detection, Lanthanides, Bacteria, Polymers, Sensors

**Supporting Information**

1. **Materials and methods**

Tween20, TOPO, obtained from TCI. The bacterial strains in this work (DSM 1116, *Escherichia coli*; DSM 90, *Bacillus megaterium*) were purchased from DSMZ (Leibniz Institute DSMZ; German Collection of Microorganisms and Cell Cultures GmbH, Braunschweig, Germany). Emission and excitation spectra as well as luminescence decay of the dispersions were measured on an Edinburgh FS5 spectrofluorometer at room temperature. Particle sizes were determined by dynamic light scattering with a Microtrac Nanotrac Wave II Q particle analyzer. The photoreactor TAK120 LC was used for the irradiation of rose bengal for ^1^O_2_ generation. Microscopy images were taken with a Leica DMi8 fluorescence microscope equipped with filtercubes (Eu^3+^: excitation 365 nm, emission 612 nm) and a CoolLED pE-4000 LED light source. The pictures were taken using 100-fold magnification objectives; the irradiance intensity was set to 30%. The camera exposure time was 130 ms.

1. **Calculation of beads-to-bacteria ratio**

The number of bacteria cells or colony forming units (CFU) per mL was determined by growing dilutions of the dispersion on tryptic soy agar plates and then counting the number of CFU observed after 24 hours incubation at 36 °C. Using the concentration of polymer beads in the bacteria dispersions, the densities of polystyrene (1.057 g/cm^3^)^1^ and polymethylmethacrylate (1.2 g/cm^3^)^2^ and, the measured mean particle sizes of the synthesized PS_Eu and PMMA_Eu probes, the average number of the NP’s in dispersion could be determined as well.

1. **FTIR spectra**

**
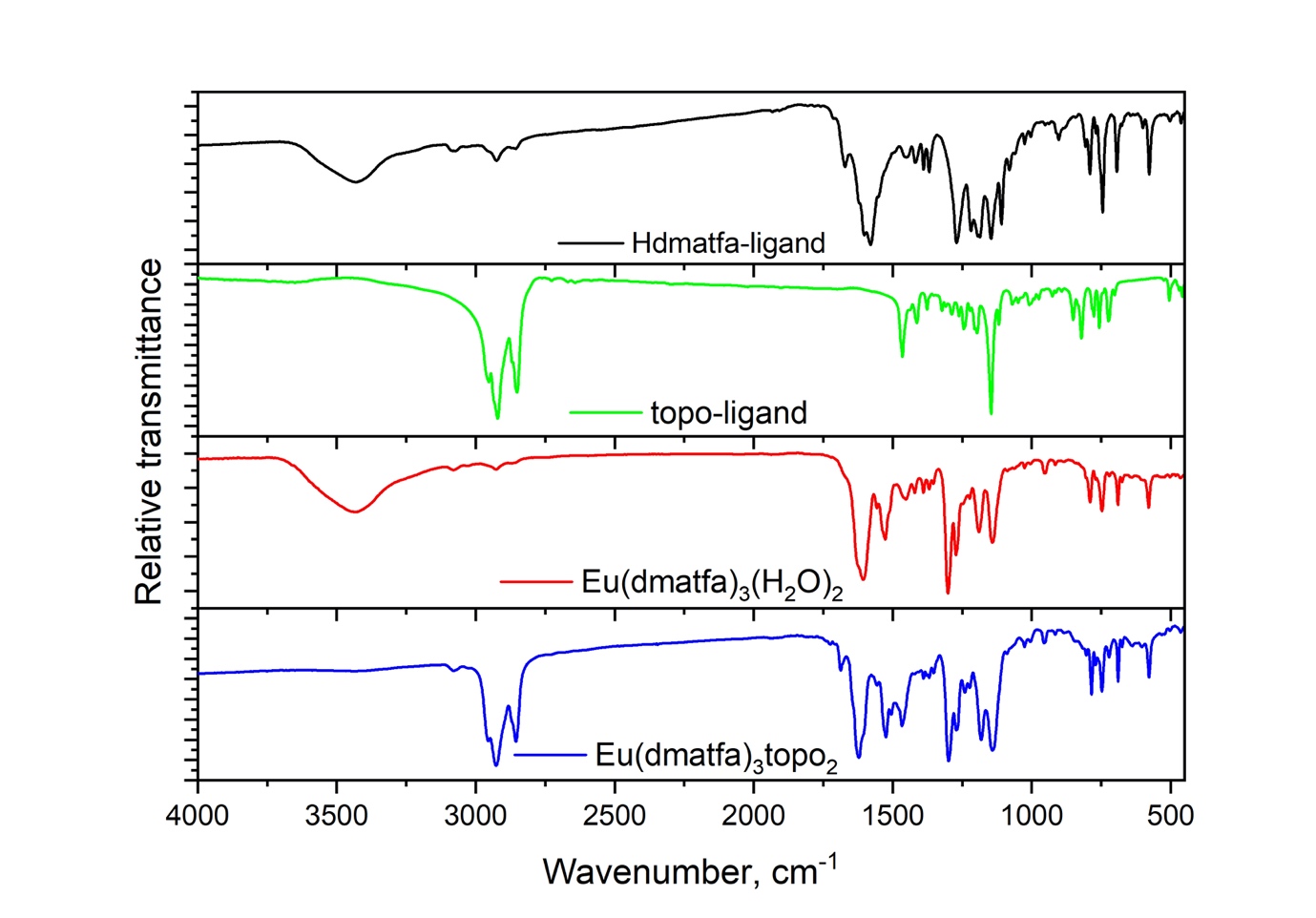
**

The disappearance of coordinated water from Eu(EP-dmatfa)_3_(H_2_O)_2_ due to its replacement by topo-ligands is clearly evident from the absence of OH-vibrations above 3000 cm^-1^.

1. **Elemental analysis of Eu(dmatfa)_3_topo_2_**

| Carbon | theoretical | 66.34% |
| --- | --- | --- |
|  | experimental | 68.45% |
| Hydrogen | theoretical | 7.37% |
|  | experimental | 7.89% |
| Europium | theoretical | 7.77% |
|  | experimental | 8.21% |

1. **Photoluminescence quantum yield (PLQY) and lifetime of the endoperoxidized complexes in toluene (degassed with Ar)**

| Complex | PLQY | Decay time, average value @ 614 nm [µs] |
| --- | --- | --- |
| Eu(**EP**-dmatfa)_3_(H_2_O)_2_ | 0.09 ±0.02 | 387 ±1.4 |
| Eu(**EP**-dmatfa)_3_topo_2_ | 0.19 ±0.02 | 588 ±0.9 |

1. **References**

(1) Pugh, T. L.; Heller, W. Density of Polystyrene and Polyvinyltoluene Latex Particles. *J. Colloid Sci.* **1957**, *12* (2), 173–180. https://doi.org/https://doi.org/10.1016/0095-8522(57)90004-1.

(2) Ali, U.; Karim, K. J. A.; Buang, N. A. A Review of the Properties and Applications of Poly (Methyl Methacrylate) (PMMA). *Polym. Rev.* **2015**, *55*, 678–705.
